# Early Ultrafast Ultrasound Imaging of Cerebral Perfusion correlates with Ischemic Stroke outcomes and responses to treatment in Mice

**DOI:** 10.7150/thno.44233

**Published:** 2020-06-12

**Authors:** Vincent Hingot, Camille Brodin, Florent Lebrun, Baptiste Heiles, Audrey Chagnot, Mervé Yetim, Maxime Gauberti, Cyrille Orset, Mickael Tanter, Olivier Couture, Thomas Deffieux, Denis Vivien

**Affiliations:** 1 Institute Physics for Medicine Paris, Inserm U1273, ESPCI Paris, CNRS FRE 2031, PSL University.; 2 Normandie Univ, UNICAEN, INSERM, GIP Cyceron, Institut Blood and Brain @Caen-Normandie (BB@C), UMR-S U1237, Physiopathology and Imaging of Neurological Disorders (PhIND), Caen, France.; 3 CHU Caen, Department of radiology, Caen University Hospital, Avenue de la Côte de Nacre, Caen, France.; 4 CHU Caen, Department of Clinical Research, Caen University Hospital, Avenue de la Côte de Nacre, Caen, France.; 5 STROK@LLIANCE, ETAP-Lab, 2 rue des Rochambelles, Caen, France

**Keywords:** Ischemic stroke, Thrombolysis, Ultrasound Imaging, Ultrasound Localization Microscopy, Outcome

## Abstract

In the field of ischemic cerebral injury, precise characterization of neurovascular hemodynamic is required to select candidates for reperfusion treatments. It is thus admitted that advanced imaging-based approaches would be able to better diagnose and prognose those patients and would contribute to better clinical care. Current imaging modalities like MRI allow a precise diagnostic of cerebral injury but suffer from limited availability and transportability. The recently developed ultrafast ultrasound could be a powerful tool to perform emergency imaging and long term follow-up of cerebral perfusion, which could, in combination with MRI, improve imaging solutions for neuroradiologists.

**Methods:** In this study, in a model of *in situ* thromboembolic stroke in mice, we compared a control group of non-treated mice (N=10) with a group receiving the gold standard pharmacological stroke therapy (N=9). We combined the established tool of magnetic resonance imaging (7T MRI) with two innovative ultrafast ultrasound methods, ultrafast Doppler and Ultrasound Localization Microscopy, to image the cerebral blood volumes at early and late times after stroke onset and compare with the formation of ischemic lesions**.**

**Results:** Our study shows that ultrafast ultrasound can be used through the mouse skull to monitor cerebral perfusion during ischemic stroke. In our data, the monitoring of the reperfusion following thrombolytic within the first 2 h post stroke onset matches ischemic lesions measured 24 h. Moreover, similar results can be made with Ultrasound Localization Microscopy which could make it applicable to human patients in the future.

**Conclusion:** We thus provide the proof of concept that in a mouse model of thromboembolic stroke with an intact skull, early ultrafast ultrasound can be indicative of responses to treatment and cerebral tissue fates following stroke. It brings new tools to study ischemic stroke in preclinical models and is the first step prior translation to the clinical settings.

## Introduction

Cerebral arterial recanalization and tissue reperfusion are the major prognostic factors of good functional outcomes following ischemic stroke. The only FDA approved pharmacological treatment of stroke remains thrombolytic therapy, using recombinant tissue plasminogen activator (rtPA), with a therapeutic window of 4.5 h after stroke onset 1. The Extend clinical trial suggests the safe use of rtPA alone, even 9 h after stroke onset 2. The overall acute recanalization rate after rtPA treatment is below 35 %, with its efficacy affected by the time to treatment, poor collaterals, clot localization and the size of thrombi 3, 4. This efficacy is dramatically improved when combined with endovascular thrombectomy (EVT) when treated within 6-24 h of the onset of symptoms 5, 6.

The main imaging techniques dedicated to brain hemodynamics are positron emission tomography (PET), single photon emission computed tomography (SPECT), Xenon-enhanced computed tomography (XeCT), dynamic perfusion computed tomography (PCT), MRI dynamic susceptibility contrast (DSC), arterial spin labeling (ASL) and transcranial Doppler ultrasonography (TCD). Finer estimation of cerebral perfusion can be obtained in Perfusion Weighted Imaging (PWI) but requires the injection of MR contrast agents and only provide one reading 7. Currently, there are no tools that could be performed at bedside (as TCD), repeatable (as ASL), provides quantitative measurement (as PET and XeCT) and measures multiple perfusion parameters (as PCT) 8,9. The recent development of ultrafast ultrasound and progresses in probe technologies combined all these criteria in a unique system 10. Unlike optical methods that are used for cerebral perfusion imaging, ultrasound allows the imaging in depth in living tissues. Specifically, ultrafast Doppler allows the monitoring of subtle Cerebral Blood Volume (CBV) changes without contrast agents and led to the development of the ultrasound analog of functional MRI (fMRI): functional Ultrasound (fUS) 11-16. Ultrafast ultrasound also allows the detection of injected intravascular microbubbles, a clinical ultrasound contrast agent, and led to the development of Ultrasound Localization Microscopy (ULM) 17-20 which, unlike ultrafast Doppler, may allow transcranial imaging in adults patients 21,22. In this study, we demonstrate that both modalities are adapted to the study of stroke in a mouse model, with ultrafast Doppler producing longitudinal monitoring and ULM proving an increased sensitivity and definition, two criteria which are mandatory for clinical applicability.

In preclinical studies where the skull was removed, ultrafast Doppler have been showed to detect vessels with flow as slow as 1 mm.s^-1^ with a 100 µm precision whereas ULM detected vessels under 1 mm.s^-1^ with a 10 µm resolution. In this study, the skull was kept intact and image quality was degraded but still enabled quality imaging of cerebral perfusion. Our present work demonstrates in an intact skull setup with a clinically relevant model of Middle Cerebral Artery (MCA) occlusion 23-25 that ultrafast Doppler and ULM can provide characterization of cerebral perfusion during an ischemic episode and follow-up in mice. The potential of ULM for transcranial imaging in human patients might allow the imaging of cerebral perfusion very early after stroke onset and possible improvement in medical care.

## Materials and Methods

### Animals

Experiments were performed on swiss male mice (35-40 g; Janvier Labs, France, 8-10 weeks old) in accordance with French ethical laws (Decree 2013-118) and European Communities Council guidelines (2010/63/EU).

### Middle Cerebral Artery occlusion in the thromboembolic model

Micropipettes were filled with 1 µL of purified murine alpha thrombin (1 UI = 0.05 mg; Stago BNL). The pipettes were introduced in the lumen of the MCA and murine thrombin was slowly injected to form a fibrin clot. Micropipettes were left in place for 10 min to stabilize the clot.

### Tissue-Type Plasminogen Activator induced thrombolysis

To induce thrombolysis, 10 mice received intravenous injection of 200 µL of rtPA (10 mg/kg, Actilyse), 10% as a bolus and 90% as an infusion for 40 min at a steady rate of 4.5 μL/min. A control group of 10 mice was injected with saline under similar conditions. One animal was excluded from the rtPA group due to poor sterotactic fixing.

### Ultrafast ultrasound

**Figure 1:**
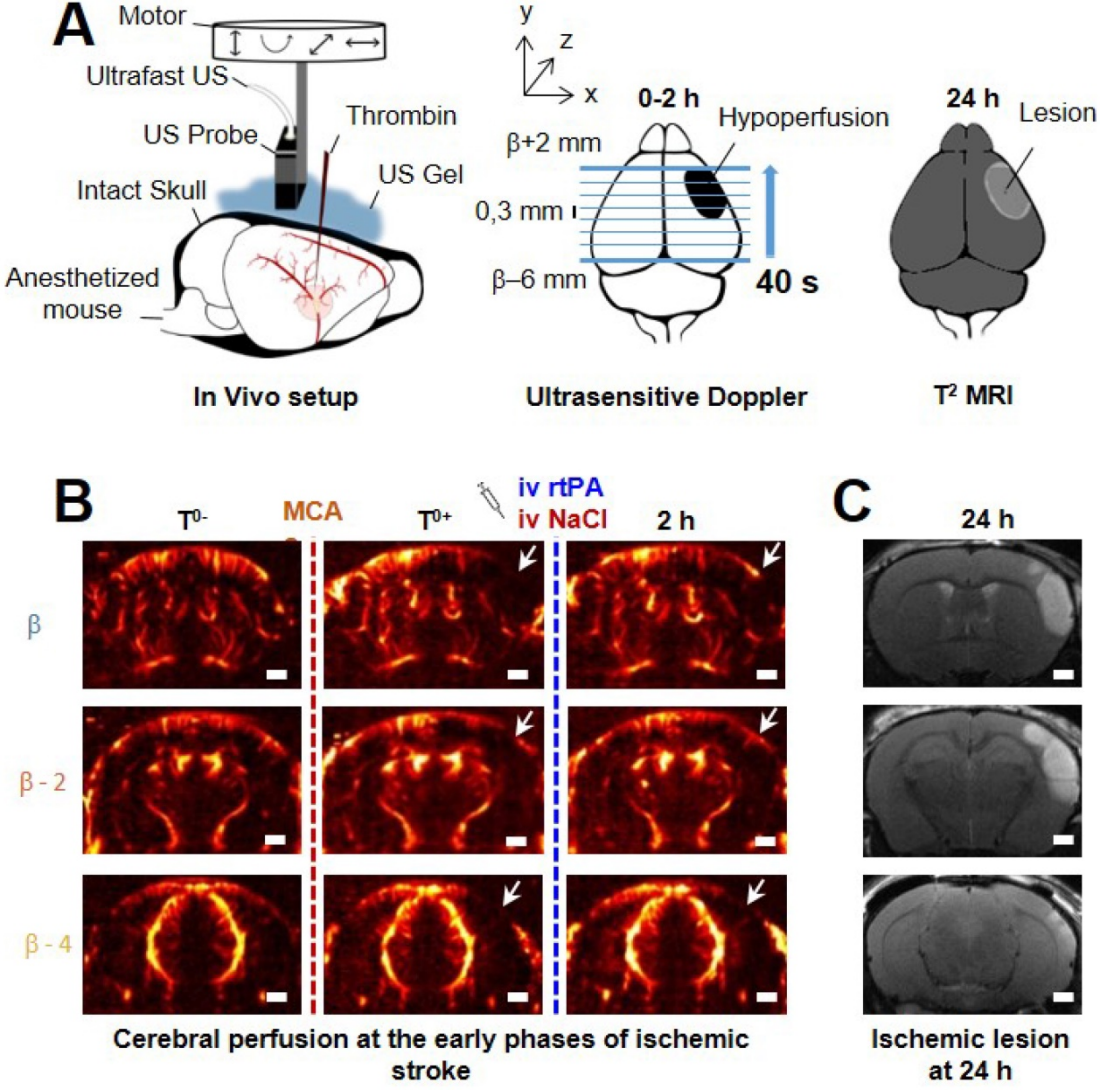
**Transcranial ultrafast ultrasound imaging to monitor cerebral perfusion before, during and after stroke A**. Experimental setup with an ultrasound probe connected to an ultrafast ultrasound acquisition system (Inserm Accelerator of Technological Research) and mounted on a 4 axis-motor for 3D scanning over the whole mice brain by steps of 0.3 mm. A volume over the whole brain is reconstructed every 40 s. Ultrafast Ultrasound monitors cerebral perfusion during the early phase of the ischemic episode, before, during and after onset, including treatment with the gold standard recombinant-tPA (rtPA). At 24 h MRI reveals the final ischemic lesion. **B**. Ultrafast Doppler reveals hypoperfusion in the ipsilateral cortices subjected to thrombin injection (clot formation) in the middle cerebral artery (MCA) and reperfusion of the corresponding territory after injection of the thrombolytic, rtPA. **C**. Registration and comparison with MRI reveal tight relationships between cerebral perfusions at the early phase of stroke and the final lesion volumes. Scale bar 1 mm.

Acquisitions were performed on an ultrafast scanner (Verasonics, 128 channels, 62.5 MHz sampling rate) with Neuroscan live acquisition software (ART Inserm U1273 & Iconeus; Paris, France) with a custom ultrasound probe (15 MHz, 0.11 mm pitch, 128 elements, 14 mm width, Vermon, France) which enables a 110 μm x 100 μm in plane resolution at a depth of 10 mm. The probe was mounted on 4 motors (3 translation + 1 rotation, Pi, Germany). A schematic of the setup can be found in Figure 1A. As both stereotactic frame and motorization system were fixed to the table, a common coordinate system was set so each animal could be imaged later in similar configuration. In this study, the coordinate system is (*z, x, y*) with *z* the axial axis, *x* the lateral axis and *y* the elevation axis as shown in Figure 1A. The coordinate system was adapted to match stereotactic coordinates by taking a reference on the antero-posterior axis at the vertical plane under the Bregma suture β=0.

### Ultrafast Doppler

200 compounded frames (11 angles between -10°: 10°) were acquired at 500 Hz. Singular Value Decomposition filters were used (removal of the 60 first singular values) to separate blood signal from tissues and summed to produce a power Doppler image 26-30. Between two images, a 1.2 s pause was added to let the motor move to the next slice. 24 coronal planes were imaged every 0.3 mm to reconstruct a 10 mm × 14 mm × 8 mm volume between β+2 mm and β-6 mm with an in plane resolution of 110 µm × 100 µm and a step of 300 µm every 40 s (Figure 1B).

### Temporal profiles

Two regions of interest were defined on the body of the MCA and on the hypoperfused part of the cortex. The mean Doppler intensity in the ROI was calculated at each time to produce temporal profiles proportional to cerebral blood volumes in the ROI. The profiles were normalized on the basis of pre-occlusion levels.

### Ultrasound Localization Microscopy

To perform ULM, acquisition and post processing steps were adapted from the reference methods 17-20. For each image, 100 µL of Sonovue microbubbles were injected in the tail vein. Blocks of 800 compounded frames (-5° 0° 5°) at 1 kHz were acquired for 800 ms and saved for 200 ms and this scheme repeated for 180 s. A combination of Butterworth high pass filter (second order, 20 Hz) and SVD filters (removal of the 10 first singular values) were used to separate microbubbles echoes from tissues. Microbubbles centroid positions were localized using a weighted average algorithm. Microbubbles were tracked through consecutive frames using *simpletracker* (Mathworks). Tracks were interpolated and smoothed using a sliding window of 5 points and cleaned from redundant positions. A density image was reconstructed on an 11 µm × 10 µm grid.

### Magnetic Resonance Imaging

Acquisitions were performed on a 7T Brucker system. T2-weighted images were acquired using a multislice multiecho sequence: TE/TR 33 ms/2500 ms and reconstructed with a 0.7 mm × 0.7 mm × 0.5 mm resolution. Lesions were manually segmented on T2 acquisitions. MRI and ultrafast Doppler volumes were registered on anatomical similarities using *imregister* (Mathworks), an intensity-based function for multimodal registration. As the coordinate system was the same for ultrafast Doppler and ULM, the volumetric registration used for ultrafast Doppler was applied to ULM to ensure registration with MRI. Segmentation of ischemic lesion was performed manually and blind to ultrasound images.

### Quantifications and statistical analyses

To account for tissue swelling due to the edema, a first correction factor had to be calculated for every mouse. On T_2_ MRI images, the distance between the skull and the corpus callosum was measured on both hemisphere of the mouse brain. Because skull thickness increases at the front of the head, ultrasound imaging in the most anterior parts of the head suffered from stronger wave attenuation. Consequentially, the contrast and sensitivity to the vasculature was strongly impaired and meaningful analysis could not be performed reliably after β+1.5 mm. As lesions spread up to β+3 mm, the parts between β+1.5 mm and β+3 mm were not included in lesion quantifications in the analysis in Figure 5 under the mention adjusted volume of lesion.

Quantifications are expressed as mean ± std (Figure 5A and 5B). Statistical analysis was performed using GraphPad®. We first assessed normal distribution of all samples by Shapiro-Wilk tests. In the panels A, B and E unpaired two-tailed t tests were performed. Pearson correlation tests were used for panels C and D. Sidak's multiple comparisons tests were used to assess multiple comparisons in panel F. Differences were considered statistically significant for a probability value p < 0.05.

### Ultrafast ultrasound TICI score

In human, the TICI score is used to describe the perfusion following stroke 34,35. We adapted a TICI-like score for ultrafast Doppler. The scoring was estimated by an operator from a combination of markers: recanalisation and reperfusion profiles reaching 50% or pre-occlusion levels, and the presence of significant remaining hypoperfused volumes at 2 h. Grade 0: No Recanalisation. Grade 1: Recanalisation but no Reperfusion Grade 2: Recanalisation and partial reperfusion. Grade 3: Complete recanalisation and reperfusion.

## Results

### Transcranial ultrafast ultrasound monitors hypoperfusion following thromboembolic stroke

On ultrafast Doppler, the body of the MCA can be observed on a coronal slice over 1 mm. Directly after thromboembolic occlusion of the MCA (Figure 1A), blood flow in the artery is completely blocked and the appearance of a large hypoperfused area can be observed in the ipsilateral cortex to the occluded MCA and spreading between β -3 mm and β -4 mm (Figure 1B). In the lateral part of the cortex, fed by the MCA, the blood supply is completely stopped. The most central part of the cortex, fed by the Anterior Cerebral Artery (ACA) is sometimes subjected to small and rapid changes in cerebral blood volumes (CBVs). During the 2 h following MCAo and rtPA treatment, restoration of perfusion can be assessed. At 24 h post-stroke onset and treatment, T_2_ weighted MRI reveals areas with high water content corresponding to the ischemic lesion (Figure 1C). The edema as seen on T_2_ MRI is known to correlate with tissue damage seen by histopathological staining 23,24. Moreover, the edema only appears several hours after the stroke onset which makes it ineffective to image tissue damage during the early phase of stroke (Figure S1).

### Without rtPA treatment, the ischemic lesion is the hypoperfused volume measured early in ultrafast ultrasound

**Figure 2:**
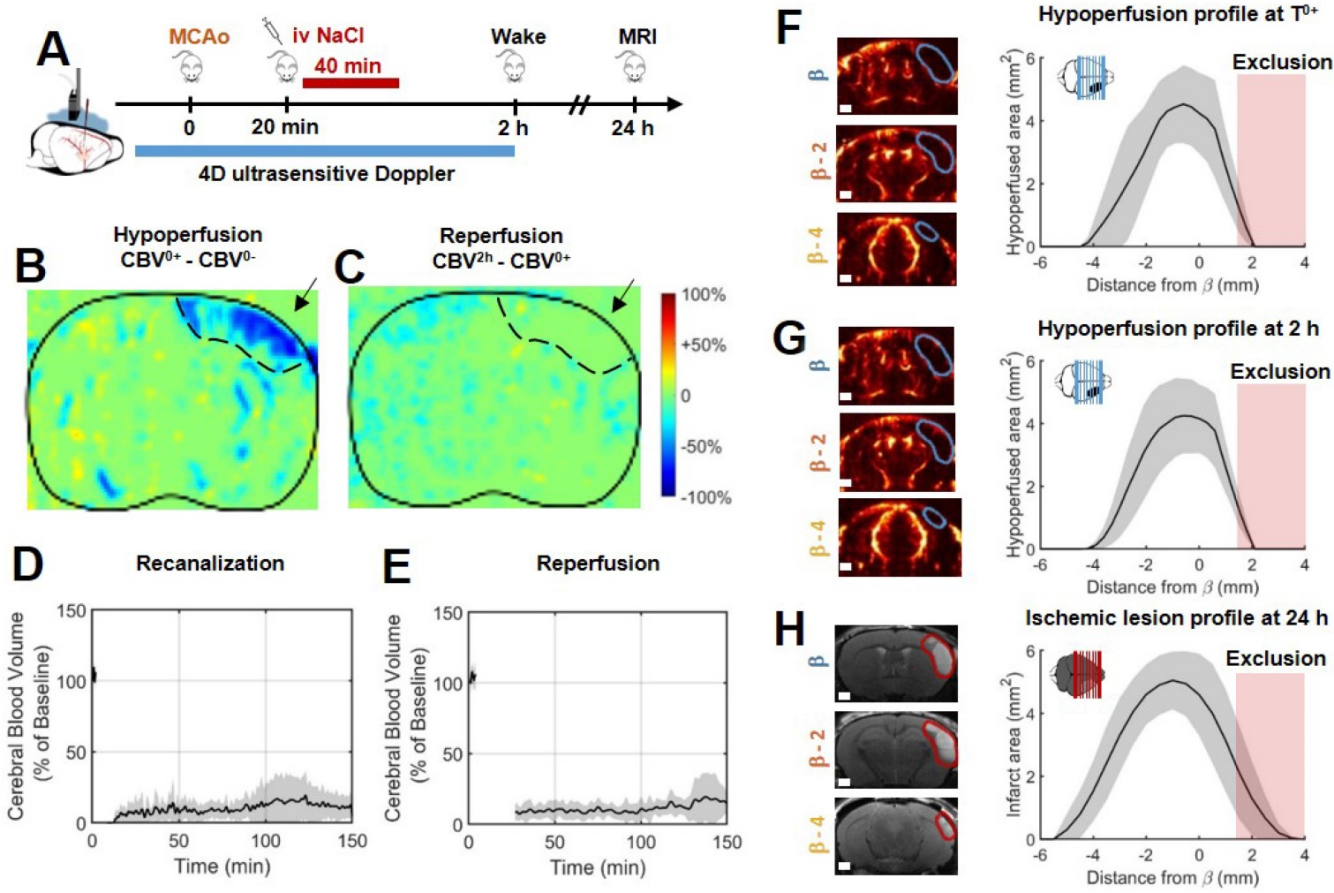
**In the absence of treatment, the final volume of lesion corresponds to the early hypoperfused area. A**. Experimental timeline for ultrasound monitoring of cerebral blood volumes after MCAo and comparison with MRI.** B**. Differences between ultrafast Doppler imaging performed before and after occlusion of the middle cerebral artery (MCAo) reveal hypoperfusion in the corresponding ipsilateral cortices. **C**. Differences between ultrafast Doppler imaging just after MCAo and 2 h later show no sign of reperfusion in the corresponding ipsilateral cortices. **D**. Monitoring of blood flows in the MCA on ultrafast Ultrasound over β+1 mm shows permanent occlusion with no clear recanalization occurring during the first 2 h. **E**. Monitoring of cerebral perfusion in the hypoperfused volume shows no sign of reperfusion. **F**. Patterns of hypoperfusions directly after MCAo reveal large hypoperfused volumes in the cortex. **G**. Patterns of hypoperfusions 2 h after MCAo reveal similar hypoperfused volumes. **H**. Patterns of the final ischemic lesions measured at 24 h after MCAo revealed by T_2_ MRI.

In the control group, 10 mice were injected with saline 20 min after MCAo, corresponding to the sham of the rtPA-treated group (Figure 2B). Variations in ultrafast Doppler before and after occlusion exhibit hypoperfusion in the corresponding ipsilateral cortices (Figure 2B). Additionally, differences in ultrafast Doppler between post-occlusion time and 2 h after the stroke onset is displayed in Figure 2C and reveal the absence of any reperfusion. After identification of the MCA on ultrafast Doppler image, recanalization profiles were plotted and exhibited the absence of recanalization for saline treated mice (Figure 2D). Deep in the tissues fed by the MCA, no reperfusion was observed in any of the mice (Figure 2E). Profiles directly after MCAo can be reconstructed and reveal a peak around β-1 mm and spreading between β-4 mm and further than β+3 mm (Figure 2F). Total measured volumes at risk were 16 mm^3^ ± 3 mm^3^. Similar measurements were performed 2 h post MCAo and show little evolutions and hypoperfused volumes stayed similar within the margin of error (Figure 2G). Infarct areas on T_2_ weighted MRI reveal lesion spreading between β-5 mm and β+3 mm with a peak also around β-1 mm and co-localization with the hypoperfused areas (Figure 2H). To account for late edema and ultrasound blindness between β+1 mm and β+3 mm, two corrections were made to the volume of lesions with adjusted volumes of 17 mm^3^ ± 3 mm^3^ (24 mm^3^ ± 5 mm^3^ without correction). This represents a 6% difference between ultrasound and MRI. In these conditions, the lesions measured by MRI at 24 h appear to be the totality of the hypoperfused volumes measured by ultrafast Doppler at 2 h post stroke onset. This hypothesis was validated in a supplementary group of 5 mice in which the MCA has been permanently occluded through electrocoagulation thus allowing neither recanalization nor reperfusion of the tissue (Figure S2). As expected, no reperfusion was observed, and similar hypoperfused volumes were observed in ultrafast Doppler imaging together with similar ischemic lesions on T_2_ weighted MRI. The volumes at risk were 19 mm^3^ ± 3 mm^3^ while the volumes of lesion were 18 mm^3^ ± 5 mm^3^ (24 mm^3^ ± 7 mm^3^ before adjustments). This represents a 5 % difference between ultrasound and MRI.

### rtPA induces recanalization and reperfusion can be monitored transcranially with ultrafast ultrasound

**Figure 3:**
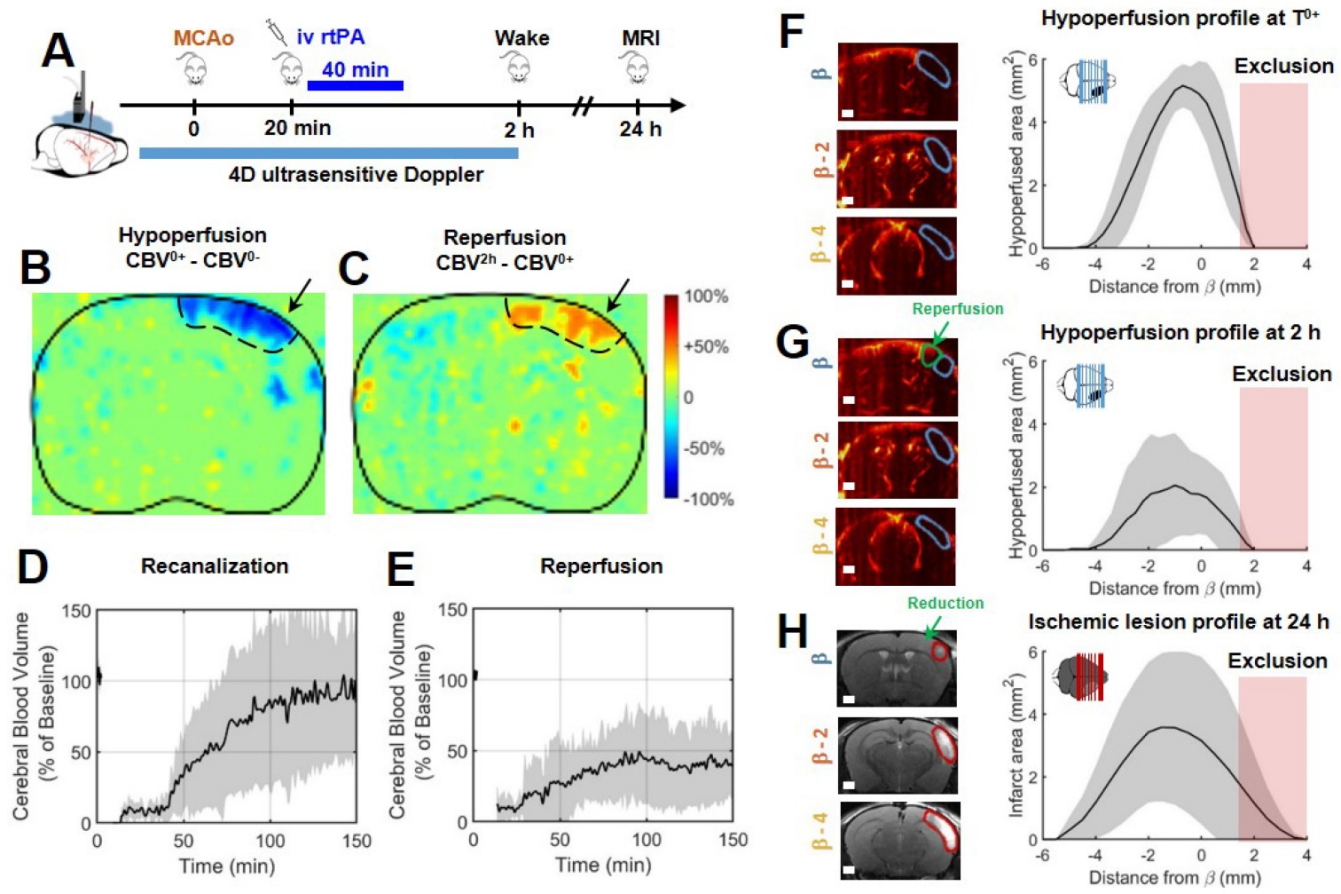
** Early injection of rtPA causes arterial recanalization, tissue reperfusion and reduces the volume of lesion. A**. Differences between ultrafast Doppler before and after MCAo reveal hypoperfusion in the corresponding ipsilateral cortices. **B**. Differences between ultrafast Doppler just after MCAo and 2 h later show reperfusion in the hypoperfused part of the corresponding cortices. **C**. Corresponding MRIs reveal the formation of smaller lesions. **D**. Monitoring of flows in the MCA on ultrafast ultrasound images over β+1 mm shows rapid and effective recanalization. **E**. Monitoring of cerebral perfusion in the hypoperfused volumes show some tissue reperfusions. **F**. Patterns of hypoperfusions directly after MCAo revealed by ultrafast ultrasound imaging.** G**. Patterns of hypoperfusions 2 h after MCAo revealed by ultrafast ultrasound imaging. **H**. Patterns of the ischemic lesions 24 h after MCAo revealed by MRI in rtPA treated animals.

In a group of 9 mice, the gold standard fibrinolytic treatment, rtPA was injected intravenously 20 min after occlusion of the MCA, 10 % in bolus and 90 % in infusion as in clinic (Figure 3A). The differences of ultrafast Doppler images from before and after MCAo exhibit the hypoperfusion in the ipsilateral cortices (Figure 3B) whereas the differences between just after occlusion and 2 h after this time display the reperfused areas (Figure 3C**)**. The monitoring of the recanalization of the body of the MCA reveals an overall improvement in arterial recanalization (Figure 3D). Although tissue reperfusion can be observed, it does not appear as effective as arterial recanalization (Figure 3E). The areas at risk in ultrafast Doppler were measured as previously (sham control group, Figure 2) directly after MCAo (before rtPA treatment) and reveal the same behaviors, a peak around β-1 mm and spreading between β-4 mm and further than β+1 mm with total hypoperfused volumes of 17 mm^3^ ± 3 mm^3^ (Figure 3F). 2 h post MCAo however, the action of rtPA treatment can be quantified as the hypoperfused volumes are reduced to 7 mm^3^ ± 7 mm^3^ (Figure 3G). In agreement with recanalization-reperfusion of the ischemic brain tissue, revealed by a rescue of the CBV, the final infarct areas on T_2_ weighted MRI at 24 h are 11 mm^3^ ± 3 mm^3^ (14 mm^3^ ± 11 mm^3^ before adjustment), (Figure 3H) i.e. reduced by 30 % of the lesion volumes compared to the initially identified areas at risk measured before rtPA treatment. There remains a 30 % difference between ultrasound and MRI which highlights the fact that although they are reperfused, some tissues can still be infarcted. The variability in the response to rtPA treatment comes from the thromboembolic model and is rather representative of what happens in human patients. This variability of behaviors can be more finely observed with ultrafast ultrasound.

### Transcranial ultrafast ultrasound allows a fine spatiotemporal follow-up of the cerebral blood volumes following stroke and treatment

**Figure 4:**
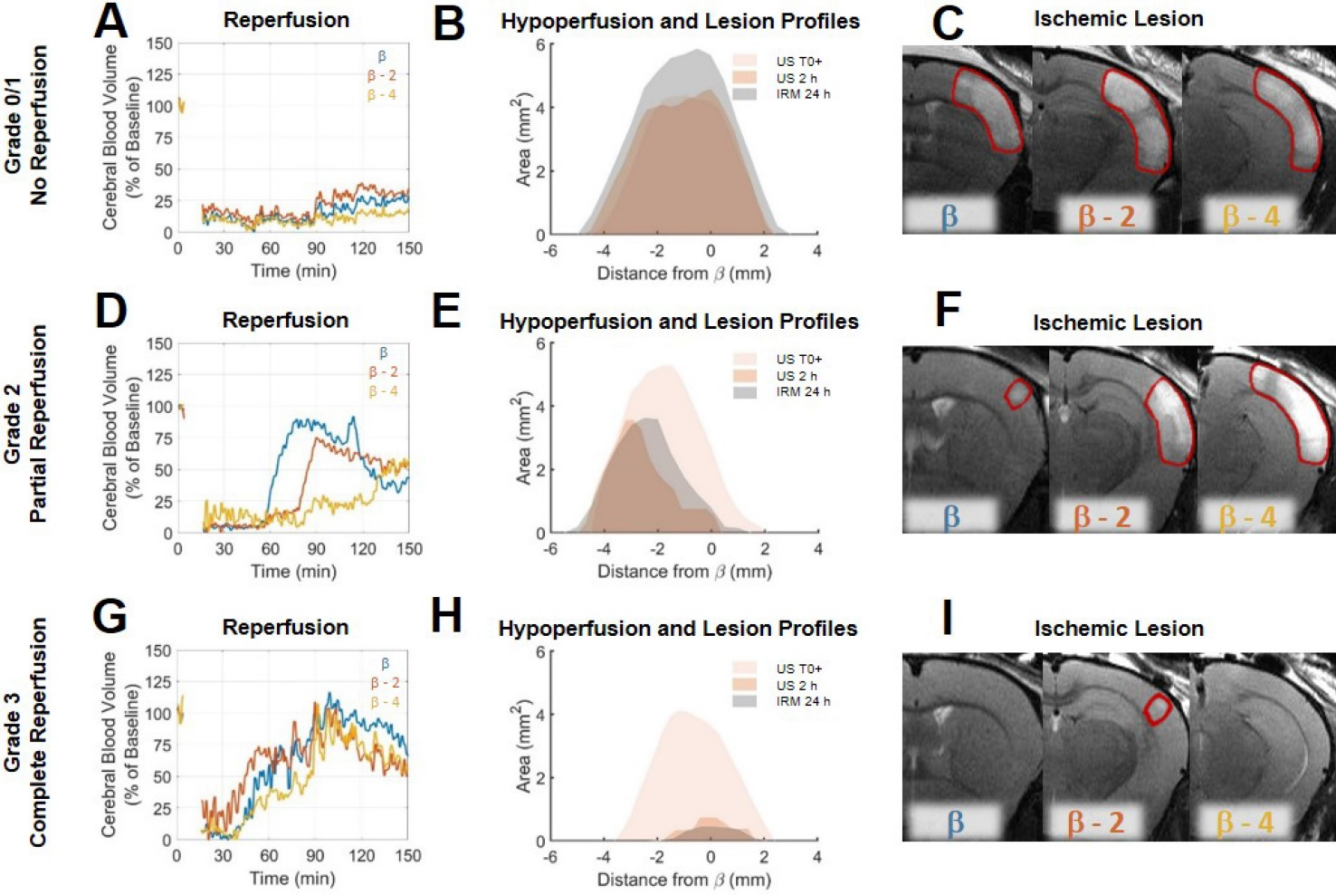
Differences in reperfusion patterns revealed by ultrafast ultrasound imaging correspond to the formation of ischemic lesions. A. For a typical Grade 0/1 mouse, the reperfusion curves in three different parts of the hypoperfused areas show no reperfusion. B. Hypoperfused profiles immediately post MCAo and after 2 h show no significant evolution of the formation of the ischemic lesions co-localized with hypoperfusions. C. Ischemic lesions on the corresponding areas. D. For a typical Grade 2 mouse, the reperfusion curves in three different parts of the hypoperfused areas show inhomogeneous reperfusions. E. Hypoperfused profiles immediately post MCAo and after 2 h show significant evolutions of the formation of the ischemic lesions co-localized with the remaining hypoperfusions at 2h. F. Ischemic lesions on the corresponding areas. G. For a typical Grade 3 mouse, the reperfusion curves in three different parts of the hypoperfused areas show early and effective reperfusions. H. Hypoperfused profiles immediately post MCAo and after 2 h show significant reductions of hypoperfusions associated with the formation of reduced ischemic lesions. I. Ischemic lesions on the corresponding areas.

Analysis of individual animals of the rtPA treated group, unmasks fine correspondence between reperfusion patterns and volumes of lesion. In two animals, some degree of arterial recanalization was observed without any tissue reperfusion. In two other animals, complete tissue reperfusions were achieved. In the other animals, recanalization was followed by some form of incomplete reperfusions. To highlight these differences and how ultrafast ultrasound can be used to understand lesion formation, reperfusion profiles were reconstructed in three different coronal planes to explore spatial temporal differences in reperfusion. To standardize the analysis of reperfusion, a grading system similar to the clinical TICI grading system 31,32 (Thrombolysis In Cerebral Ischemia) from 0 to 3 was used to describe the reperfusion, 0 being no recanalization, 1 being recanalization but no reperfusion, 2 being a partial reperfusion and 3 being a complete reperfusion. This scoring was performed based on the ultrafast Doppler images. On Figure 4 are given three examples of mice with different reperfusion patterns. The first mouse showed little arterial recanalization and no tissue reperfusion corresponding to a TICI grade of 1. The whole hypoperfused volume directly after MCAo remains hypoperfused for the duration of the experiment as shown by reperfusion curves at β (in blue), β-2 (in red) and β-4 (in yellow) (Figure 4A). The hypoperfusion profiles show no significant evolution 2 h after MCAo and the lesion is the entire hypoperfused volume (Figure 4B). MRI reveals a large lesion over the hypoperfused areas (Figure 4C). The second mouse showed good arterial recanalization but inhomogeneous tissue reperfusion corresponding to a TICI grade of 2. Some parts of the hypoperfused volume directly after MCAo are reperfused within the first 2 h as shown by reperfusion curves at β, β-2 mm and β-4 mm (Figure 4D). The hypoperfusion profiles show significant reduction 2 h after MCAo and the lesion matches the remaining hypoperfused volume (Figure 4E). MRI reveals the inhomogeneity of the lesion (Figure 4F). The third mouse showed good arterial recanalization and good tissue reperfusion corresponding to a TICI grade of 3. All the hypoperfused volume directly after MCAo is reperfused within the first 2 h as shown by reperfusion curves at β, β-2 mm and β-4 mm (Figure 4G). The hypoperfusion profiles show a complete reduction 2 h after MCAo and the absence of lesion (Figure 4H). MRI reveals the absence of lesion (Figure 4I). Eventually, the prediction ability of ultrafast ultrasound can be tested on all the animals.

### Transcranial ultrafast ultrasound at 2 h predicts responses to thrombolytic treatment and final outcomes

**Figure 5:**
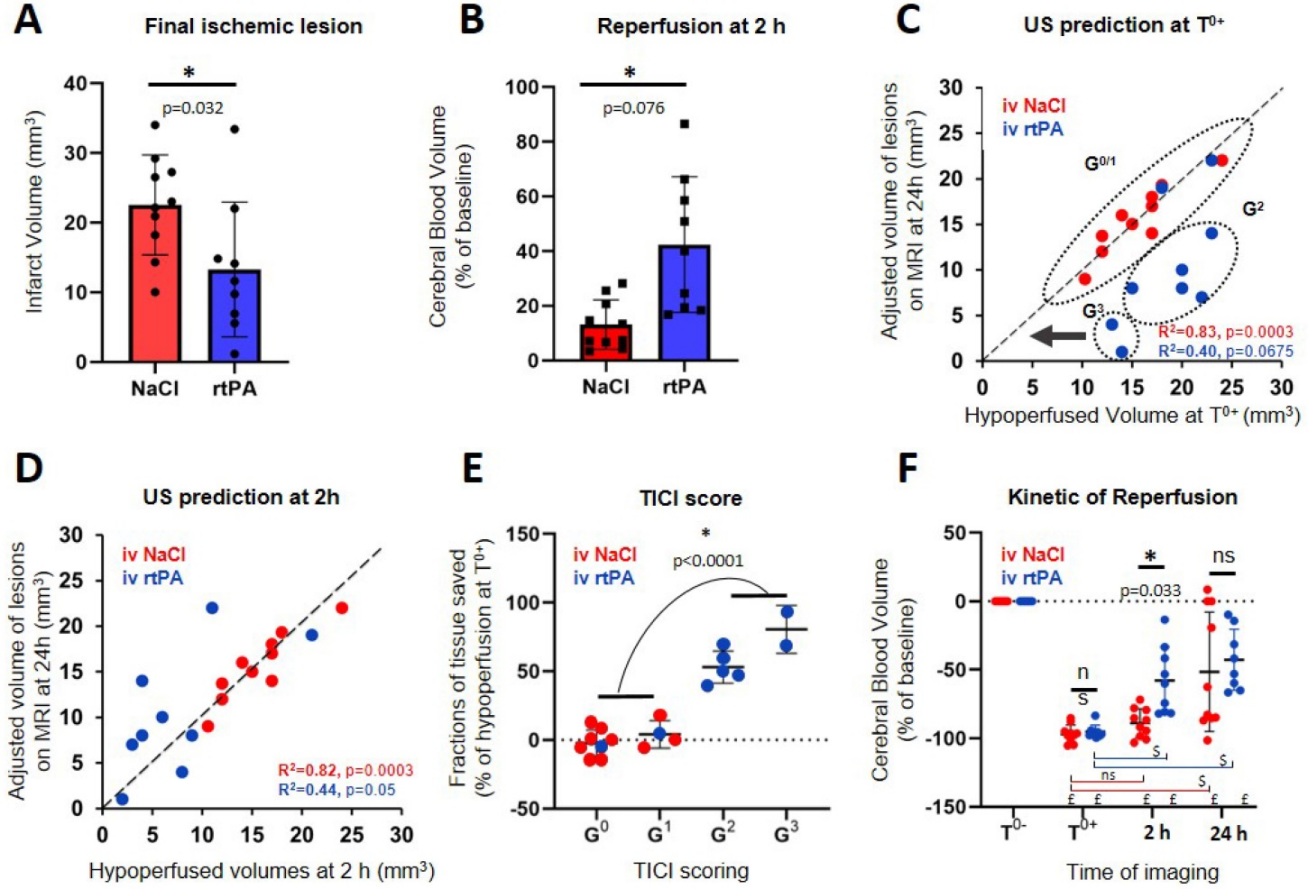
**Prediction of lesions and responses to treatment based on early Transcranial ultrafast Doppler imaging**. A, B, E = Unpaired t test Two-tailed (n=10; 9); C, D = Pearson correlation (n=10; 9); F = Sidak's multiple comparisons test (n=10; 9), *Intergroup differences; $ Intragroup differences; £ significant compared to T^0-^. A. Final ischemic lesions determined at 24 h indicate reduction of lesions in animals treated with rtPA. **B**. State of reperfusion 2 h after MCAo shows improved reperfusions in the rtPA treated group. **C**. Directly after MCAo, the hypoperfused volume in ultrafast Doppler imaging is a marker of final ischemic lesion volumes for the NaCl treated animals (red) with the lesions that correspond to the complete hypoperfused volumes, aligned around the diagonal. For the rtPA treated group (blue), the lesions are smaller than the volumes at risk defined directly after occlusion because of effective tissue reperfusions. **D**. After rtPA treatment, the remaining hypoperfused volumes are predictive of the final lesion volumes in both groups. **E**. Effectiveness of the prediction of lesions based on reperfusion using the ultrafast ultrasound based TICI score. **F.** Evolutions of perfusions in the volumes at risk at the early time after stroke onset (0 and 2 h) and at 24 h show that after a therapeutic window of 2 h, perfusion is no longer predictive of the lesion.

The effect of rtPA in this model reduces the lesion by more than 30 % (P=0.032) (Figure 5A). The saving of tissue caused by early reperfusion can be observed in rtPA animals (P=0.0206) (Figure 5B). To represent the link between hypoperfusion and the formation of a lesion, the adjusted volumes of lesion can be plotted as a function of the volumes at risk defined as the hypoperfusion directly after the occlusion of the MCA (Figure 5C). For the control group, the outcome of the stroke can be predicted since, after adjustments, all points align along the diagonal (R^2^=0.8273, p=0.0003). This means that when no early reperfusion is observed, the final volumes of lesion are the whole volumes at risk measured by using CBV only, just after stroke onset. For animals receiving rtPA, the volumes of lesion are reduced compared to the volumes at risk, meaning that tissue areas were effectively saved (R^2^=0.4001, p=0.0675). The size of the lesion depends on two factors: the initial size of the hypoperfused zone and the efficiency of the reperfusion. When plotting the adjusted volumes of lesion as a function of the hypoperfusion at 2 h after stroke onset (control and treated groups) (Figure 5D), all points align around the diagonal (NaCl: R^2^=0.8186, p=0.0003) (rtPA: R^2^=0.4438, p=0.05) with a strong correlation, even in the rtPA group with a large variability of outcome. This means that in both cases, the volumes of lesion are the hypoperfused volumes measured at 2 h using CBV only.

However, this representation does not consider the quality of the reperfusion in saving tissues. All mice in the control group are attributed a TICI score of 0 or 1, meaning that no reperfusion was ever observed. Four mice in the rtPA treated group were attributed TICI scores of 0 or 1 meaning that thrombolysis was not effective enough to reperfuse the tissues. In these conditions, the prognostic of lesions is similar to the control saline group. Six mice in the rtPA group were attributed TICI scores of 2 or 3, meaning that reperfusions were achieved. In these conditions, the lesions were reduced compared to the volumes at risk and compared to the control group, implying that the better the reperfusion is, the more tissues are saved. Using the ultrafast Doppler based TICI grading system, we can represent how much tissue was effectively saved by an early and effective reperfusion compared to the initial areas at risk measured by CBV (Figure 5E). It is no surprise that for grades 0 and 1, no tissue is saved. On the contrary, major improvements can be observed for mice with grade 2 and 3 with preservation of more than half of the tissue (P<0.0001). All animals were imaged again at 24 h. Some reperfusion 24 h after the onset of the stroke is observed in both the saline and rtPA groups, with an improved reperfusion for the rtPA treated animals when measured at 2 h (p=0.033) (Figure 5F).

### Transcranial Ultrasound Localization Microscopy to image hypoperfusion

**Figure 6:**
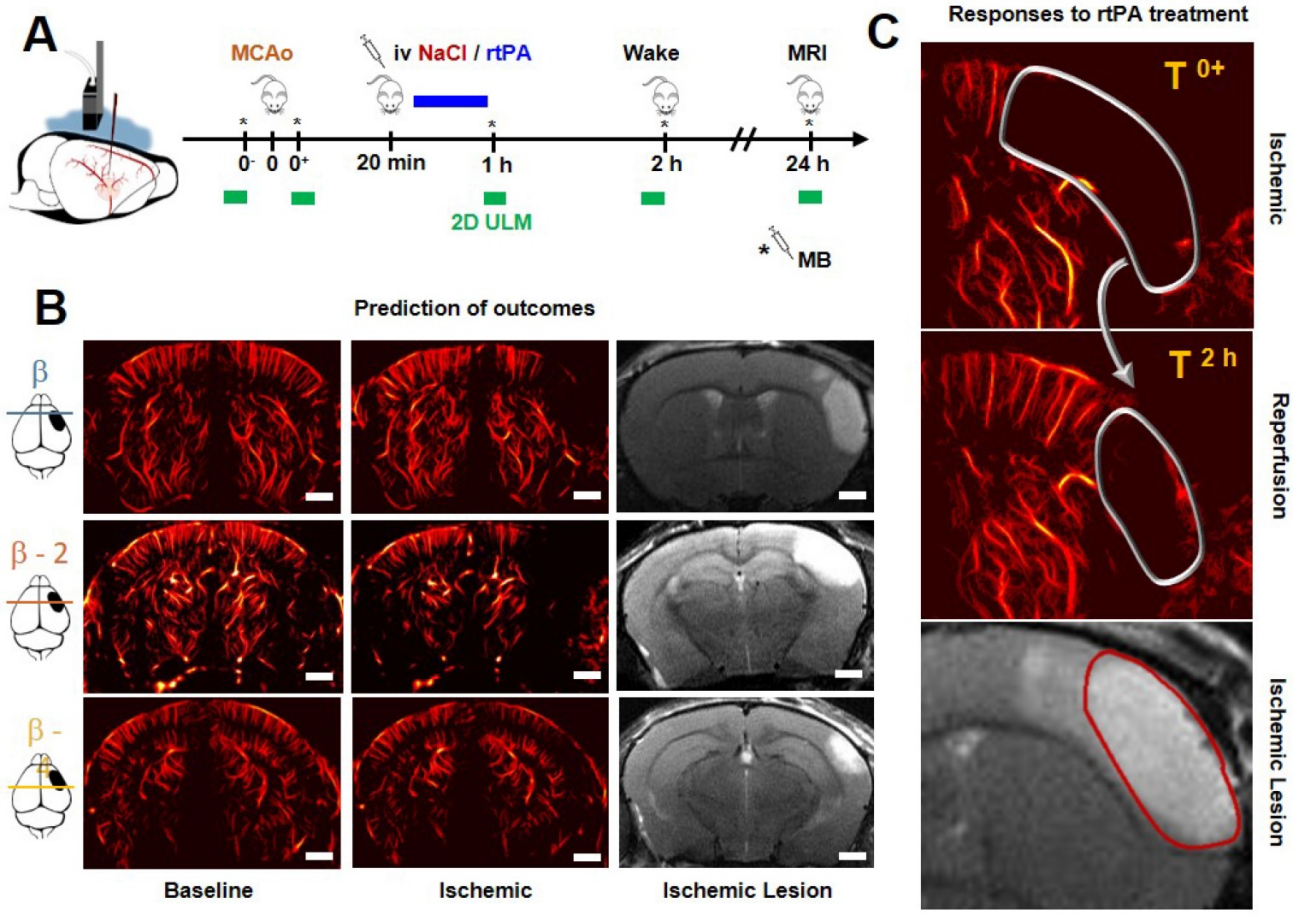
**Predicting the outcomes and evaluating responses to treatment with transcranial ULM**. **A**. Timeline for ULM acquisitions. **B**. For mice treated with NaCl, a large lesion can be seen in ULM just after ischemia and very little reperfusion can be observed 2 h after the onset. In ultrafast Doppler, the hypoperfused area is the infarcted lesion seen in MRI. **C.** On rtPA treated mice, the reperfusion can be evaluated with ULM and predicts the formation of the lesion. Scale bar 1 mm.

In four mice in each group, ULM was performed in a single coronal plane at particular times, just before and after occlusion of the MCA, 1 and 2 h later and again after 24 h (Figure 6A). On the given examples in Figure 6B, although there is still shadowing by the skull, ULM can be performed and reveals the vasculature with enhanced spatial resolution and sensitivity. ULM reveals the localization and extent of the hypoperfusion. 24 h after the onset of the stroke, we can compare the lesions seen in T_2_ weighted MRI with the hypoperfused areas. The given examples in Figure 6B, shows the formation of a large lesion. The hypoperfused area is large to begin with, and the injection of NaCl does not induce efficient reperfusion. 2 h after the onset of the ischemia, only a small area is reperfused, which we can easily identify as saved on the corresponding T_2_ image. The given example in Figure 6C shows the formation of a reduced lesion after rtPA treatment and reperfusion. These examples give an idea of how ULM could be used to image the hypoperfused areas and monitor the response to treatment.

## Discussion

An important goal to improve stroke therapy is to identify how the lesion will extend with time, whether the patient will respond to treatment and ultimately to predict the functional outcome. To address these important questions, neurologists and neuroradiologists use a combination of prediction tools with the severity of stroke, its location, age, previous risk factors and comorbid disease (such as high blood pressure, diabetes, etc.) as key factors. Despite all these criteria, their answer remains most often very evasive and is usually postponed until the evolution of permanent neurological deficits. An early biomarker to accurately identify salvageable tissues and to predict functional outcome after stroke is, therefore, mandatory 33 and could be met with the assistance of neuroimaging techniques 34.

In our study, we demonstrate that ultrafast Doppler and Ultrasound Localization Microscopy can be relevant neuroimaging modalities for stroke. We show here that early assessment of the cerebral perfusion using *in vivo* ultrafast Doppler brain imaging, when performed early after stroke onset (here 2 h in rodents) is indicative of final lesion volumes measured by MRI at 24 h. We also demonstrate, that when performed just after thrombolytic treatment (rtPA-induced fibrinolysis), the methodology can be used to monitor the treatment efficacy.

Ultrafast Doppler for functional imaging was demonstrated in different animal models 11-14 and in clinical settings: in newborns 15 or intraoperatively 16. In the context of stroke, it was first investigated in 2012 35 and recent works confirmed its relevance 36,37, but not in a model of thromboembolic stroke as it occurs in clinic and without considering prognostic and/or responses to the gold standard treatment. However, the translation of results to human should always be done carefully, especially in the context of stroke. Indeed, all strategies identified so far to save the brain excepted rtPA treatment have failed in the clinic, mainly because of poor animal modeling. The model of stroke we have used here 23,24 is relevant with the actual clinical setting 25,38. Moreover, ultrasound imaging of the early phases following stroke was also performed with optical contrast using photoacoustic imaging but in a simpler mouse model of photo thrombose and filament induced MCA occlusion 39. Unlike ultrafast Doppler and ULM, photoacoustic imaging is sensitive to oxygen saturation in tissue and not blood volumes but can nonetheless provide similar results in terms of temporal dynamics and formation of lesion 40. Although its translation to human patients might be severely limited by the strong absorption of light through the skull, photoacoustic can be complementary with ultrafast Doppler and ULM for preclinical studies to provide both cerebral blood volume and tissue oxygenation in tissues with different sets of sensitivity and resolutions.

Similarly, the translation of ultrasound imaging methods to human has always been a challenge because of a strong attenuation by the skull bone. As demonstrated previously, ultrafast Doppler can be performed through the fontanel 15 and could thus contribute to the diagnosis of stroke in babies. However, there are clear limitations about the possibility to use ultrafast ultrasound in adult due to strong aberrations and absorptions by the skull bone. The more recent improvements of both contrast and resolution of ULM may allow transcranial imaging 21,22, all the more so than microbubbles are commonly used in human as contrast agent for ultrasound imaging (Sonovue, Bracco), and in particular in MCA infarction to improve the signal to noise ratio 41. This is a major point considering the recent report made by the Food and Drug Administration (FDA) about a Safety Announcement related to gadolinium-based contrast agents (GBCAs) administered for MRIs 42. Nonetheless, the quality of the imaging through the skull will be critical to determine the quality of the readings, as there is a priori no way to distinguish ischemic from shadowed areas.

A proper clinical trial has yet to demonstrate the ability of ULM to provide quality reading through the human skull. The clinical use of ULM in this context could be performed through the temporal window, and would benefit from the extension of the field of view from 2D to 3D to access large areas of the brain which may reduce the dependency of the imaging procedure to the operator and allow the detection of scattered lesion. It may also enable the estimation of quantitative biomarkers of vascular suffering over large areas of the brain. Such extension is envisioned to become possible in the next years with the advent of piezocomposite 2D Matrix Arrays or Capacitive Micromachined Ultrasonic Transducers (CMUT) technologies and development of 3D ultrafast ultrasound modalities 43-45.

The possibility to have access to an easy to use, cheap, operator independent and transportable machine to image cerebral perfusion would undoubtedly increase the number of patients eligible for treatment, with the possibility to use this imaging method in the ambulance. Although recanalization is associated with rapid clinical improvement in some patients, for others despite recanalization, they show delayed or impaired reperfusion. Ideal patient selection for thrombolysis alone or combined to thrombectomy should therefore not be based on therapeutic windows but rather on perfusion imaging to determine “a signature” of response to treatments. In addition, such type of monitoring could be very useful for long term recording of recovery and to prevent and treat recurrent stroke.

In conclusion, we provide here, in a relevant model of thromboembolic stroke model in mice with rtPA treatment, the demonstration that ultrafast ultrasound *in vivo* imaging can be used in the context of stroke to diagnose ischemic injury, to prognose outcomes and responses to treatment, in an early time frame after stroke onset.

## Supplementary Material

Supplementary figures and tables.

## References

[1] Zivin J, Fisher M, DeGirolami U, Hemenway C, Stashak J (1985). Tissue plasminogen activator reduces neurological damage after cerebral embolism. Science.

[2] Campbell BCV, Ma H, Ringleb PA, Parsons MW, Churilov L, Bendszus M (2019). Extending thrombolysis to 4·5-9 h and wake-up stroke using perfusion imaging: a systematic review and meta-analysis of individual patient data. Lancet.

[3] Berkhemer OA, Fransen PSS, Beumer D, van der Berg LA, Lingsma HF, Yoo AJ (2015). A Randomized Trial of Intraarterial Treatment for Acute Ischemic Stroke. N Engl J Med.

[4] Coutinho JM, Liebeskind DS, Slater LA, Nogueira RG, Clark W, Davalos A (2017). Combined Intravenous Thrombolysis and Thrombectomy vs Thrombectomy Alone for Acute Ischemic Stroke: A Pooled Analysis of the SWIFT and STAR Studies. JAMA Neurol.

[5] Nogueira RG, Jadhav AP, Haussen DC, Bonafe A, Budzik RF, Bhuva P (2018). Thrombectomy 6 to 24 h after Stroke with a Mismatch between Deficit and Infarct. N Engl J Med.

[6] Thiebaut AM, Gauberti M, Martinez De Lizarrondo S, Vivien D, Yepes M, Roussel BD (2018). The role of plasminogen activators in stroke treatment: fibrinolysis and beyond. Lancet Neurol.

[7] Shen Q, Duong T (2016). Magnetic resonance imaging of cerebral blood flow in animal stroke models. Brain Circ.

[8] Wintermark M, Sesay M, Barbier M, Borbely K, Dillon WP, Eastwood JD (2005). Comparative Overview of Brain Perfusion Imaging Techniques. Stroke.

[9] Essig M, Shiroishi MS, Nguyen TB, Saake M, Provenzale JM, Enterline D (2013). Perfusion MRI: The Five Most Frequently Asked Technical Questions. AJR Am J Roentgenol.

[10] Tanter M, Fink M (2014). Ultrafast imaging in biomedical ultrasound. IEEE Trans Ultrason Ferroelectr Freq Control.

[11] Deffieux T, Demene C, Pernot M, Tanter M (2018). Functional ultrasound neuroimaging: a review of the preclinical and clinical state of the art. Curr Opin Neurobiol.

[12] Mace E, Montaldo G, Cohen I, Baulac M, Fink M, Tanter M (2011). Functional ultrasound imaging of the brain. Nat Methods.

[13] Demene C, Tiran E, Sieu LA, Bergel A, Gennisson JL, Pernot M (2016). 4D microvascular imaging based on ultrafast Doppler tomography. NeuroImage.

[14] Tiran E, Ferrier J, Deffieux T, Genisson JL, Pezet S, Lenkei Z (2017). Transcranial Functional Ultrasound Imaging in Freely Moving Awake Mice and Anesthetized Young Rats without Contrast Agent. Ultrasound Med Biol.

[15] Demene C, Baranger J, Bernal M, Delanoe C, Auvin S, Biran V (2017). Functional ultrasound imaging of brain activity in human newborns. Sci Transl Med.

[16] Imbault M, Chauvet D, Gennisson JL, Capelle L, Tanter M (2017). Intraoperative Functional Ultrasound Imaging of Human Brain Activity. Sci Rep.

[17] Errico C, Pierre J, Pezet S, Dessailly Y, Lekei Z, Couture O (2015). Ultrafast ultrasound localization microscopy for deep super-resolution vascular imaging. Nature.

[18] Couture O, Hingot V, Heiles B, Muleki-Seya P, Tanter M (2018). Ultrasound Localization Microscopy and Super-Resolution: A State of the Art. IEEE Trans Ultrason Ferroelectr Freq Control.

[19] Hingot V, Errico C, Heiles B, Rahal L, Tanter M, Couture O (2019). Microvascular flow dictates the compromise between spatial resolution and acquisition time in Ultrasound Localization Microscopy. Sci Rep.

[20] Heiles B, Correia M, Hingot V, Pernot M, Provost J Tanter M (2019). Ultrafast 3D Ultrasound Localization Microscopy using a 32×32 Matrix Array. IEEE Trans Med Imaging.

[21] O'Reilly MA, Hynynen K (2013). A super-resolution ultrasound method for brain vascular mapping. Med Phys.

[22] Soulioti De, Espíndola D, Dayton PA, Pinton GF (2020). Super-Resolution Imaging Through the Human Skull. IEEE Trans Ultrason Ferroelectr Freq Control.

[23] Orset C, Haelenwyn B, Allan SM, Ansar S, Sampos F, Cho TH (2016). Efficacy of Alteplase in a Mouse Model of Acute Ischemic Stroke: A Retrospective Pooled Analysis. Stroke.

[24] Orset C, Macrez R, Young AR, Panthou D, Angles-Cano E, Maubert E (2007). Mouse Model of In Situ Thromboembolic Stroke and Reperfusion. Stroke.

[25] Llovera G, Hofmann K, Roth S, Salas-Perdomo A, Ferrer-Ferrer M, Perego C (2015). Results of a preclinical randomized controlled multicenter trial (pRCT): Anti-CD49d treatment for acute brain ischemia. Sci Transl Med.

[26] Demene C, Deffieux T, Pernot M, Osmanski BF, Gennisson JL, Sieu LA (2015). Spatiotemporal Clutter Filtering of Ultrafast Ultrasound Data Highly Increases Doppler and fUltrasound Sensitivity. IEEE Trans Med Imaging.

[27] Baranger J, Arnal B, Perren F, Baud O, Tanter M, Demene C (2018). Adaptive Spatiotemporal SVD Clutter Filtering for Ultrafast Doppler Imaging Using Similarity of Spatial Singular Vectors. IEEE Trans Med Imaging.

[28] Shung KK, Sigelmann R, Reid JM (1976). Scattering of ultrasound by blood. IEEE Trans Biomed Eng.

[29] Cloutier G, Qin Z (1997). Ultrasound backscattering from non-aggregating and aggregating erythrocytes-a review. Biorheology.

[30] Bercoff J, Montaldo G, Loupas T, Savery D, Meziere F, Find M (2011). Ultrafast compound Doppler imaging: providing full blood flow characterization. IEEE Trans Ultrason Ferroelectr Freq Control.

[31] Zaidat OO, Lazzaro MA, Liebeskind DS, Janjua N, Wechsler L, Nogueira RG (2012). Revascularization grading in endovascular acute ischemic stroke therapy. Neurology.

[32] Zerna C, Thomalla G, Campbell BCV, Rha JH, Hill MD (2018). Current practice and future directions in the diagnosis and acute treatment of ischaemic stroke. Lancet.

[33] Stinear CM (2017). Prediction of motor recovery after stroke: advances in biomarkers. Lancet Neurol.

[34] Thomalla G, Gerloff C (2019). Acute imaging for evidence-based treatment of ischemic stroke. Curr Opin Neurol.

[35] Martin A, Mace E, Boisgard R, Montaldo G, Theze B, Tanter M (2012). Imaging of perfusion, angiogenesis, and tissue elasticity after stroke. J Cereb Blood Flow Metab.

[36] Brunner C, Isabel C, Martin A, Dussaux C, Savoye A, Emmrich J (2017). Mapping the dynamics of brain perfusion using functional ultrasound in a rat model of transient middle cerebral artery occlusion. J Cereb Blood Flow Metab.

[37] Brunner C, Korostelev M, Raja S, Montaldo G, Urban A, Baron JC (2018). Evidence from functional ultrasound imaging of enhanced contralesional microvascular response to somatosensory stimulation in acute middle cerebral artery occlusion/reperfusion in rats: A marker of ultra-early network reorganization?. J Cereb Blood Flow Metab.

[38] Martinez de Lizarrondo S, Gakuba C, Herbig BA, Repesse Y, Ali C, Denis CV (2017). Potent Thrombolytic Effect of N -Acetylcysteine on Arterial Thrombi. Circulation.

[39] Lv J, Shi L, Jingde Z, Fei D, Zhiyou W, Ronghe C (2020). In vivo photoacoustic imaging dynamically monitors the structural and functional changes of ischemic stroke at a very early stage. Theranostics.

[40] Cao R, Li J, Kharel Y, Zhang C, Morris E, Santos WL (2018). Photoacoustic microscopy reveals the hemodynamic basis of sphingosine 1-phosphate-induced neuroprotection against ischemic stroke. Theranostics.

[41] Seidel G, Meyer-Wiethe K, Berdien G, Hollstein D, Toth D, Aach T (2004). Ultrasound Perfusion Imaging in Acute Middle Cerebral Artery Infarction Predicts Outcome. Stroke.

[42] Levine D, McDonald RJ, Kressel HY (2018). Gadolinium Retention After Contrast-Enhanced MRI. JAMA.

[43] Provost J, Papadacci C, Arango JE, Imbault M, Fink M, Genisson JL (2014). 3D ultrafast ultrasound imaging in vivo. Phys Med Biol.

[44] Provost J, Papadacci C, Demene C, Gennisson JL, Tanter M, Pernot M (2015). 3-D ultrafast Doppler imaging applied to the noninvasive mapping of blood vessels in vivo. IEEE Trans Ultrason Ferroelectr Freq Control.

[45] Rabut C, Correia M, Finel V, Pezet S, Pernot M, Deffieux T (2019). 4D functional ultrasound imaging of whole brain activity in rodents. Nat Methods.

