# Supplementary material for: Early Ultrafast Ultrasound Imaging of Cerebral Perfusion correlates with Ischemic Stroke outcomes and responses to treatment in Mice
Source: Theranostics. 2020 Jun 12;10(17):7480–91. doi: 10.7150/thno.44233 (PMC7359089; doi:10.7150/thno.44233)
Supplement: Supplementary file 1 — Supplementary figures and tables. [file thnov10p7480s1.pdf]

Supplementary Materials and Methods

Animals: Experiments were approved by institutional review board (French ministry of Research) and local ethical committee of Normandy (CENOMEXA) registered under the reference CENOMEXA-C2EA – 54 and received the agreement number #13412.

Surgical procedures: Mice were anesthetized with isoflurane (induction 5 % and maintenance 2 %) in a gas mixture of N_20/O_2 (70/30 %). Once placed in a stereotactic frame, a catheter was placed in the tail vein, to allow intravenous injection. Although not essential, the skin above the skull was opened and pushed to the sides of the head to limit the impact of air bubbles potentially trapped in hairs. Internal mice temperature was maintained at 37 °C using a rectal probe and a heating pad. A 1 cm skin incision between the ear and eye was performed. The masseter muscle was retracted, a local craniotomy was done on the temporal side of the skull to expose the middle cerebral artery (MCA) and the dura was removed locally in preparation for MCA occlusion. Ultrasound gel was placed on the mouse skull. After 2 h of ultrasound imaging, the skin was stitched and sprayed liberally with lidocaïne. 24 h later, mice were anesthetized, placed on the stereotactic frame, catheterized, and their skin opened before another session of ultrasound imaging and sacrific.

Middle Cerebral Artery occlusion in the electrocoagulation model: The electrosurgical coagulator (AESCULAP Typ TB 50 Electrosurgical Unit Bipolar Coagulator) was used. The bipolar mode was selected at 8W. The artery was coagulated under MCA bifurcation between its posterior and anterior branches (distal occlusion) with the forceps and was cut transversely to ensure the success of the permanent occlusion surgery.

Image processing: The 4D matrix was first reshaped in a 2D Casorati matrix before singular value decomposition was applied. A combination of singular values were formed on the basis of similarities of the temporal vectors. Spatial vectors were reshaped to a 3D volume highlighting the regions exhibiting the corresponding temporal behavior. Delimitation of the areas was done using the *watershed* technique (Mathworks), for morphological segmentation. Temporal profiles could then be computed as the average intensity in the segmented region of interest. The identification of the ischemic areas is therefore done without any prior knowledge.

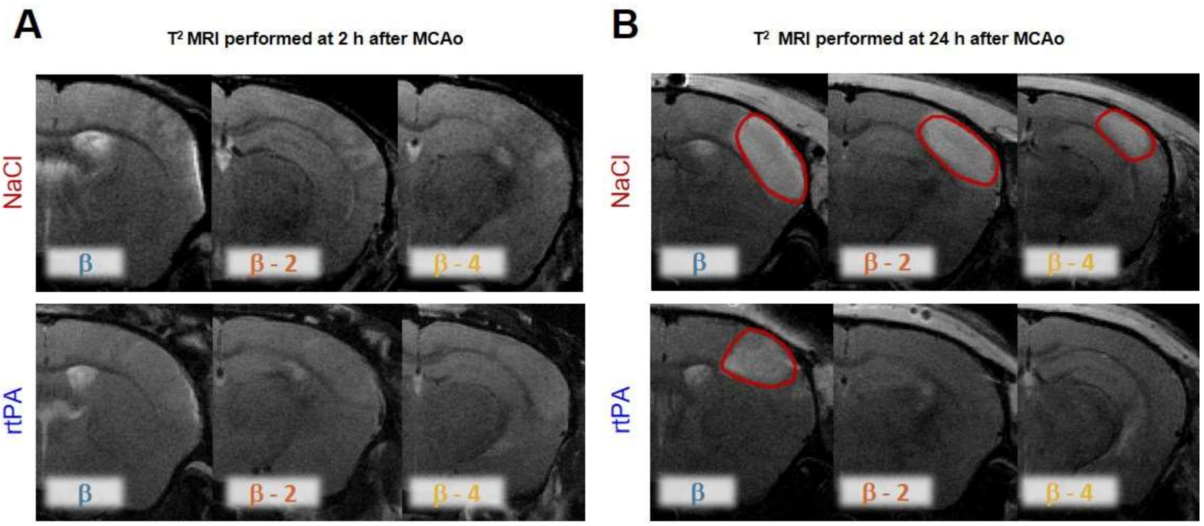

Supplementary Figure 1. Dynamic of ischemic lesions formation in T2 MRI. A. Representative coronal views of T2 MRI in NaCl and rtPA treated groups 2 h post MCA occlusion showing no visible lesion in any group and no difference between groups. B. Representative coronal views of T2 MRI in NaCl and rtPA treated groups 24 h post MCA occlusion showing visible lesions for the control group and clear differences between NaCl and rtPA treated animals.

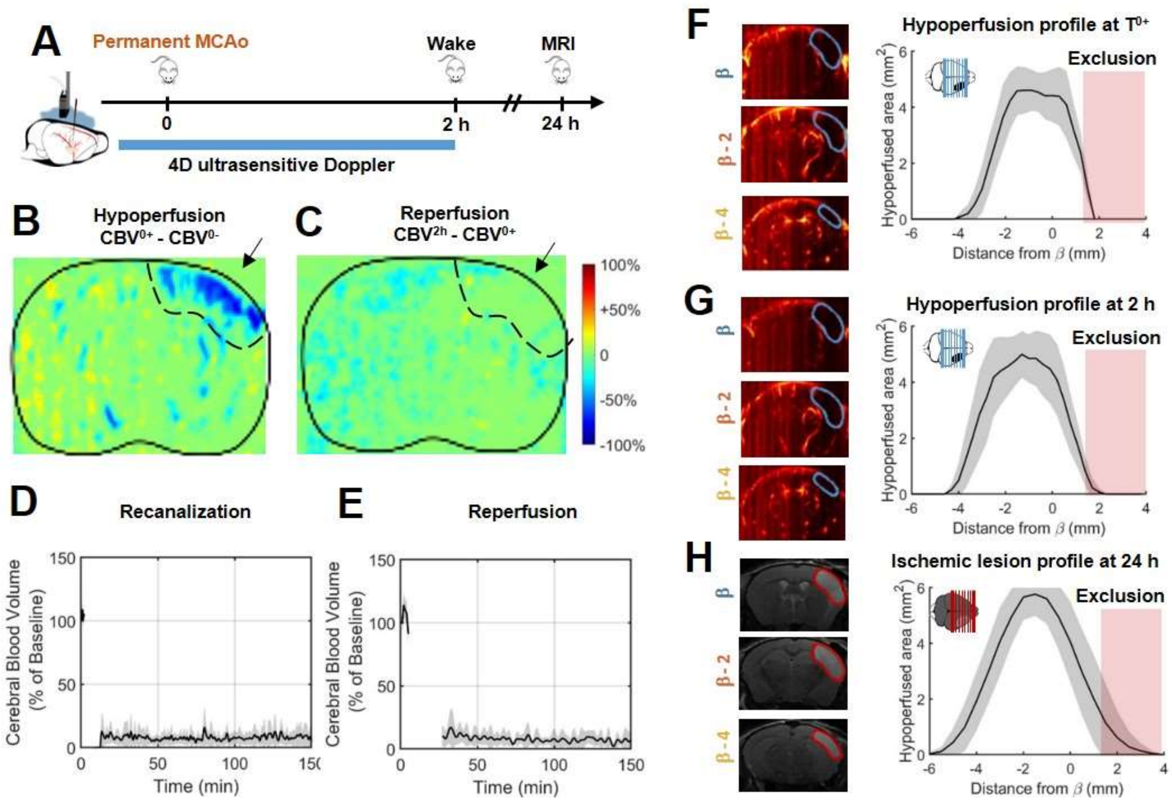

Supplementary Figure 2. Formation of ischemic lesions in an electrocoagulation model of stroke. A. Differences between ultrasensitive Doppler before and after MCAo reveal hypoperfusion in the corresponding ipsilateral cortices. **B**. Differences between ultrasensitive Doppler just after MCAo and 2 h later show no significant reperfusion. **C**. Corresponding MRIs reveal the formation lesions. **D**. Monitoring of flow in the MCA on Ultrafast Ultrasound images over β+1 mm shows no recanalization. **E**. Monitoring of cerebral perfusion in the hypoperfused volumes show no tissue reperfusion. **F**. Pattern of hypoperfusions directly after MCAo revealed by Ultrafast Ultrasound imaging. **G**. Pattern of hypoperfusions 2 h after MCAo revealed by Ultrafast Ultrasound imaging. **H**. Pattern of the ischemic lesions 24 h after MCAo revealed by MRI in rtPA treated animals.
